# Liver function changes after transarterial chemoembolization in US hepatocellular carcinoma patients: the LiverT study

**DOI:** 10.1186/s12885-019-5989-2

**Published:** 2019-08-13

**Authors:** Rebecca A. Miksad, Sadahisa Ogasawara, Fang Xia, Marc Fellous, Fabio Piscaglia

**Affiliations:** 1 Department of Hematology and Oncology, Beth Israel Deaconess Medical Center and Harvard Medical School, Boston, MA USA; 2 Department of Gastroenterology, Chiba University, Chiba, Japan; 3 Pharmaceutical Division, Bayer HealthCare Pharmaceuticals, Whippany, NJ USA; 4 Department of Medical and Surgical Sciences, University of Bologna General and University Hospital S.Orsola-Malpighi, Bologna, Italy

**Keywords:** Liver failure, Hepatic ischemic damage, HCC systemic therapy

## Abstract

**Background:**

The real-world incidence of chronic liver damage after transarterial chemoembolization (TACE) is unclear. LiverT, a retrospective, observational study, assessed liver function deterioration after a single TACE in real-world hepatocellular carcinoma (HCC) patients in US practice.

**Methods:**

Eligible HCC patients identified from Optum’s integrated database using standard codes as having had an index TACE between 2010 and 2016 with no additional oncologic therapy in the subsequent 3 months. At least one laboratory value (bilirubin, albumin, aspartate transaminase [AST], alanine transaminase [ALT], international normalized ratio [INR]) was required at baseline and the acute (≤29 days after TACE) and chronic (30–90 days after TACE) periods. Due to lack of universally accepted liver function deterioration criteria, clinically meaningful changes in laboratory parameters were pre-defined by authors (FP, RM, and SO).

**Results:**

Of the 3963 TACE patients, 572 were eligible for analyses. Deterioration of liver function from baseline occurred in the acute period and persisted in the chronic period (bilirubin 30 and 23%, albumin 52 and 31%, AST 44 and 25%, ALT 43 and 25%, INR 25 and 15%, respectively). In a subgroup analysis, a higher proportion of patients with diabetes had deterioration in AST and ALT.

**Conclusions:**

A clinically meaningful proportion of real-world HCC patients had deterioration of liver function-related laboratory values 30–90 days after a single TACE in modern US practice. Future electronic health record research may help determine causality. The present findings highlight the need for the careful selection of patients for TACE, which is important to help optimize the benefit of the overall HCC treatment course.

**Electronic supplementary material:**

The online version of this article (10.1186/s12885-019-5989-2) contains supplementary material, which is available to authorized users.

## Background

Transarterial chemoembolization (TACE) is a commonly used locoregional procedure that is recommended by several guidelines as a first-line treatment for patients with unresectable hepatocellular carcinoma (HCC) that is confined to the liver with no vascular invasion [1–3].

Signs of acute liver injury, such as elevation in liver enzymes and worsening of liver function tests, are commonly seen following TACE [4–7]. Although this acute deterioration (often defined as ≤30 days) is well documented, the extent to which TACE impacts mid- to long-term liver function is less clear for real-world patients; some studies have reported that acute liver damage may become chronic or irreversible [7–9].

Liver damage associated with locoregional therapies may adversely impact liver function, worsen prognosis, and limit the use of effective systemic treatment options, which have expanded over recent years [10, 11]. Due to the prominent role of TACE for HCC treatment, establishing longer term effects on liver function is important. This retrospective study aimed to assess the proportion of real-world HCC patients in the US who develop chronic deterioration of liver function after receiving a single TACE.

## Methods

### Study design and patients

LiverT, a retrospective, observational, real-world cohort study, used data from the Optum integrated database to identify eligible US patients with HCC. Consequently, all decisions of diagnostic procedures, treatment, disease management, and resource utilization were dependent on a mutual agreement between patient and physician, without interference by the study sponsor or protocol.

Data collected by Optum from January 1, 2009 to June 30, 2016 were extracted. Optum, a division of UnitedHealth Group (Minnetonka, MN), comprises a number of health data and information companies, providing an integrated database of healthcare claims data, combined with a longitudinal electronic health record database housed by Humedica. The study population included patients ≥18 years of age who previously had at least one TACE procedure and an HCC diagnosis code within 1 year prior to the index TACE (the first TACE procedure performed January 1, 2010 to March 31, 2016). The time periods were chosen to allow for at least 3 months’ follow-up after TACE.

The cohort only included patients with at least one documented liver-related laboratory parameter (Table 1) at each of the three time points: baseline (< 30 days before TACE), acute (0–29 days after TACE), and chronic periods (30–90 days after TACE). Patients were excluded if they had received TACE within 1 year prior to the index TACE and if they received any of these HCC treatments within 3 months after the index TACE: additional TACE, radiofrequency ablation, percutaneous ethanol injection, liver resection or transplantation, chemotherapy, sorafenib, or radioembolization by yttrium-90 (Y90). Patients were also excluded if Y90 radioembolization was recorded on the index date.

**Table 1:** Liver function thresholds to establish clinically meaningful deterioration after TACE in a real-world setting (primary analysis)

Parameter	Deterioration threshold (change from baseline)
Serum total bilirubin	Increase of ≥ 50%
Serum albumin	Decrease by ≥ 0.3 g/dL
AST	Increase of > 25%
ALT	Increase of > 25%
INR	Increase of ≥ 25%

*ALT* alanine transaminase, *AST* aspartate transaminase, *INR* international normalized ratio, *TACE* transarterial chemoembolization

The procedural coding used for all criteria are listed in Additional file 1: Table S1. Medical history (hepatitis B virus [HBV], hepatitis C virus [HCV], alcoholic cirrhosis, hypertension, and diabetes), disease status (portal vein thrombosis [PVT], distant metastases, presence of ascites, and encephalopathy), and prior HCC treatment were also extracted. The two types of PVT, bland and portal tumor infiltrated, could not be separately identified by different codes. Data on medical history and prior HCC treatment were available within 1 year prior to the index TACE; data on disease status were available within 30 days of the index TACE. The database included the month and date of death (when known).

Patient data were de-identified by an independent statistical expert following the Health Insurance Portability and Accountability Act of 1996 procedures and managed according to customer data use agreements.

### Outcomes and assessments

#### Outcomes

The primary endpoint was the proportion of patients treated with TACE who had clinically relevant deterioration of liver function laboratory values in the chronic period compared with baseline (Table 1).

Secondary endpoints included the proportion of patients with liver deterioration during the acute period, and liver deterioration in the acute and chronic periods according to baseline albumin–bilirubin (ALBI) grade (developed to objectively assess hepatic dysfunction), in the absence of Child–Pugh scores [12]. ALBI grades were determined by the ALBI score (log_10_ (bilirubin [μmL/L]) × 0.66) + (albumin [g/L] × (− 0.085)) and defined as grade 1 (≤ − 2.60), grade 2 (> − 2.60 to ≤ − 1.39), and grade 3 (> − 1.39). Survival status was reported, defined as the time from TACE to death from any cause. Patients alive at the last date known were censored at that date.

#### Assessments

Levels of serum total bilirubin, albumin, aspartate transaminase (AST), serum alanine transaminase (ALT), and international normalized ratio (INR) were extracted from the database. Due to lack of formally accepted criteria to measure liver deterioration in the setting of HCC treatment and limitations of currently used approaches, clinically relevant changes were pre-defined by preliminary consensus of the authors (FP, RM, and SO only) (Table 1). Simply reporting the mean worsening of the laboratory values was not felt to sufficiently describe the clinical relevance of worsening. The authors based their judgement of deterioration upon the worsening of laboratory values included in the Child–Pugh score and MELD score, the two most widely utilized scores to assess liver function. Notably, it was decided not to calculate the complete Child–Pugh score since it would be subject to a high degree of uncertainty, especially related to the lack of reported data in the assessment for the presence and severity of ascites and encephalopathy. The laboratory value closest to the index date was used for the baseline period and the worst laboratory value used for the acute period (assessed as change from baseline). To minimize the risk of overestimating long-term liver function deterioration following TACE, the last (not worst) laboratory value was used for the chronic period (compared with values from baseline and the acute period). Conversely, the worst values were selected in the acute period in order to capture the possibly largest, although transient impact that TACE had on liver function. The median number of days related to the last and worst values for the chronic period was assessed for each parameter and are given in Additional file 1: Table S2.

#### Sensitivity analysis

We performed a sensitivity analysis for bilirubin and albumin based on Child–Pugh categorization because of its prognostic importance in patients with cirrhosis (Table 2). Child–Pugh considers the potential impact of baseline levels and corresponds to the Common Terminology Criteria for Adverse Events (CTCAE) deterioration definitions.

**Table 2:** Bilirubin and albumin deterioration thresholds based on Child–Pugh categorization (sensitivity analysis)

Parameter	Deterioration thresholds
Serum total bilirubin at baseline	
< 2 mg/dL	If ≥ 2 mg/dL or an increase of 100%
≤ 2–≤ 3 mg/dL	If > 3 mg/dL
> 3 mg/dL	If increased by ≥ 1 mg/dL
Serum albumin level at baseline	
> 3.5 g/dL	If ≤ 3.5 g/dL or a decrease of ≥ 0.3 g/dL
≤ 2.8–≤ 3.5 g/dL	If < 2.8 g/dL
< 2.8 g/dL	If decreased by ≥ 0.3 g/dL

#### Exploratory analyses

Exploratory subgroup analyses of liver function deterioration were performed according to baseline Child–Pugh-based bilirubin levels (< 2, 2–3, and > 3 mg/dL for bilirubin only), etiology (HBV, HCV, and alcoholic cirrhosis), diabetes status, and in patients without PVT at baseline. INR was not evaluated here since anticoagulation use can potentially confound the results. An additional exploratory analysis was conducted to assess INR deterioration using only the patients who did not use anticoagulants.

### Statistical analysis

All variables were analyzed using descriptive statistics. Laboratory results were described in absolute values (mean and standard deviation, and median with range). The Sign Test was used to generate *P*-values testing the null hypothesis that the median difference in laboratory values between two time points (from baseline to the acute or chronic periods) is zero. Reported *P*-values should be interpreted with caution and no adjustments for multiplicity were made. The incidence of deterioration for each laboratory value from baseline to the chronic and acute periods was calculated based on the total population and reported with a 95% confidence interval (CI).

## Results

### Baseline characteristics

A total of 3963 patients received at least one TACE between January 1, 2010 and March 31, 2016 and were ≥ 18 years of age with an HCC diagnosis code within 1 year prior to index TACE. The full study eligibility criteria were met by 572 patients (14%); exclusions were primarily due to lack of required laboratory data (Additional file 1: Table S3). Most patients were male (72%) and the median age was 62 years (Table 3).

**Table 3:** Patient demographics and baseline characteristics prior to TACE

	TACE patients (*N* = 572)
Median age, years (range)	62 (20–88)
Sex, n (%)	
Male	411 (72)
Female	161 (28)
Disease characteristics, n (%)^a^	
Ascites	94 (16)
Distant metastases	36 (6)
Portal vein thrombosis	29 (5)
Encephalopathy	4 (1)
General medical history, n (%)^b^	
Hypertension	294 (51)
Diabetes	195 (34)
Potential etiology of HCC, n (%)^b^	
Alcoholic cirrhosis	123 (22)
Non-alcoholic cirrhosis	425 (74)
HCV	217 (38)
HBV	39 (7)
Viral hepatitis, unspecified	17 (3)
Prior (non-TACE) treatment of HCC, n (%)^b^	
Chemotherapy	45 (8)
Sorafenib	23 (4)
Liver resection	8 (1)
Percutaneous ethanol injection	3 (1)
Radioembolization by Y90	3 (1)
Liver transplantation	0
Radiofrequency ablation	0

^a^Diagnosis code < 30 days before index TACE, multiple diagnoses possible; ^b^< 1 year prior to index TACE, multiple responses possible

*HBV* hepatitis B virus, *HCC* hepatocellular carcinoma, *HCV* hepatitis C virus, *TACE* transarterial chemoembolization, *Y90* yttrium-90

### Liver function at baseline and after TACE

Laboratory values were available at all three time points for most patients, apart from INR, which was available for fewer patients. There was a large variation in baseline levels, ranging from normal to outside of normal ranges. In the acute period, deterioration is evident for all laboratory parameters (Table 4). Importantly, levels of AST and ALT were almost completely restored to baseline values in the chronic period, which was expected after an acute insult to the liver, such as with TACE. In contrast, albumin, INR, and bilirubin were only partially improved, remaining significantly worse compared with baseline (Table 4).

**Table 4:** Laboratory values at baseline and in the acute and chronic periods following TACE

Laboratory parameter	Patient number, n	Baseline (value closest to index TACE)	Acute period (highest value)	*P*-value acute vs baseline	Chronic period (latest value)	*P*-value chronic vs baseline
Bilirubin, mg/dL	462					
Mean (SD)		1.5 (1.1)	2.2 (2.9)	–	2.3 (4.1)	–
Median (range)		1.2 (0.09–6.9)	1.4 (0.3–37.9)	*P* < .0001	1.2 (0.2–41.3)	*P* = .008
Albumin, g/dL	442					
Mean (SD)		3.2 (0.7)	2.9 (0.7)	–	3.1 (0.7)	–
Median (range)		3.3 (1.6–4.9)	2.8 (1.0–4.8)	*P* < .0001	3.1 (1.4–4.8)	*P* < .0001
AST, U/L	446					
Mean (SD)		75.9 (61.5)	152.6 (270.4)		88.5 (187.3)	
Median (range)		62 (11–844)	82 (13–3341)	*P* < .0001	60 (9–3739)	*P* = .600
ALT, U/L	441					
Mean (SD)		59.5 (49.6)	122.1 (266.8)		62.1 (82.8)	
Median (range)		45 (9–450)	60 (7–3198)	*P* < .0001	42 (7–1122)	*P* = .08
INR	251					
Mean (SD)		1.3 (0.4)	1.6 (0.9)		1.5 (0.6)	
Median (range)		1.2 (0.9–3.2)	1.3 (0.9–6.9)	*P* < .0001	1.2 (0.9–6.9)	*P* < .0001

Acute period, 0–29 days after TACE; chronic period, 30–90 days after TACE

*ALT* alanine transaminase, *AST* aspartate transaminase, *INR* international normalized ratio, *SD* standard deviation, *TACE* transarterial chemoembolization

**Fig. 1:**
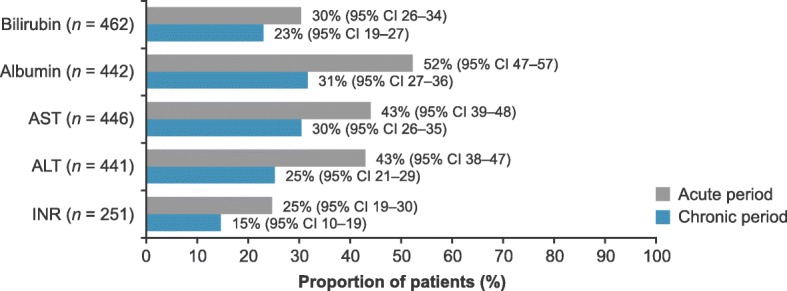
Proportion of patients with acute and chronic liver function deterioration after TACE compared to baseline (primary analysis). Acute period, 0–29 days after TACE; chronic period, 30–90 days after TACE. Deterioration thresholds: bilirubin increase of ≥ 50%, albumin decrease by 0.3 g/dL, AST increase of > 25%, ALT increase of > 25%, INR increase of ≥ 25%, all compared with baseline. *ALT* alanine transaminase, *AST* aspartate transaminase, *CI* confidence interval, *INR* international normalized ratio, *TACE* transarterial chemoembolization

In the primary analysis, although the proportion of patients with deterioration was greatest in the acute period, some still had deterioration of liver-related parameters in the chronic period (Fig. 1). This was in line with the statistically significant impact of TACE on median laboratory values (Table 4). Deterioration of bilirubin in the acute and chronic periods was observed for 30 and 23% of patients, respectively, and 52 and 31% for albumin. The sensitivity analysis using Child–Pugh-based deterioration thresholds produced similar results: bilirubin deterioration was observed in 23% of patients (*n* = 104; 95% CI 19–26) and albumin deterioration in 30% of patients (*n* = 134; 95% CI 26–35) in the chronic period.

**Fig. 2:**
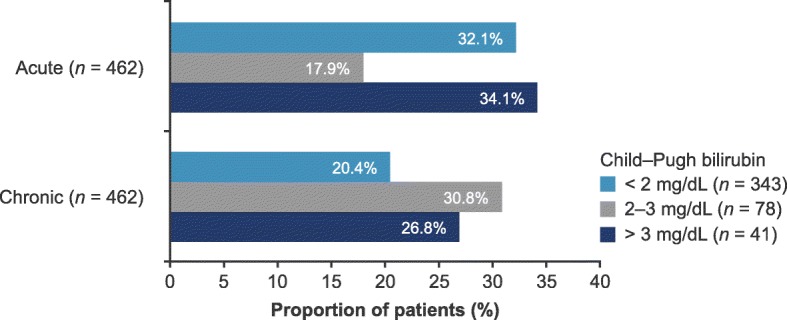
Bilirubin deterioration in acute and chronic periods after TACE using baseline Child–Pugh bilirubin categories. Acute period, 0–29 days after TACE; chronic period, 30–90 days after TACE. Deterioration threshold: bilirubin increase of ≥ 50% compared with baseline. *TACE* transarterial chemoembolization

When stratified by baseline Child–Pugh bilirubin, the proportion of patients with acute and chronic bilirubin deterioration varied following TACE (Fig. 2). For lower and upper bilirubin Child–Pugh categories, the proportion of patients with deterioration was lower in the chronic versus the acute period. However, with mild bilirubin elevation (2–3 mg/dL) bilirubin deterioration was higher in the chronic versus the acute period.

When stratified by baseline ALBI grade, there was no consistent trend in acute or chronic deterioration across liver function laboratory parameters, except for albumin. These data are shown in Additional file 1: Table S4. The proportion of patients with albumin deterioration (decrease of ≥ 0.3 g/dL) in the acute and chronic periods decreased as baseline ALBI grade increased.

Except for total bilirubin, a similar pattern of acute and chronic deterioration was seen according to HCC etiology (HBV, HCV, and alcoholic cirrhosis), the absence of PVT, and diabetes status. These analyses are given in Additional file 1: Table S5 and S6. In the chronic period, the proportion of patients with total bilirubin deterioration was lowest for patients with HBV (12%) compared with alcoholic cirrhosis (25%) and HCV (28%). The proportion of patients with INR deterioration was higher in patients on anticoagulants compared with those not on anticoagulants: 17 vs 36% and 9 vs 21% in the acute and chronic periods, respectively. These results are shown in Additional file 1: Table S7. The decrease in the proportion of patients with INR deterioration between the acute and chronic periods were similar for both groups.

### CTCAE-based definitions of deterioration

For all parameters, an ad-hoc analysis assessed the proportion of patients with deterioration according to National Cancer Institute-CTCAE (v4.03) grade, which are often used to report deterioration of liver-related parameters in clinical trials. In this analysis, the proportion of patients with acute and chronic bilirubin deterioration was 31 and 33%, respectively.

### Patient survival after TACE

At 180 days following TACE, 88 patients had died: four deaths were documented at day 30, 35 at day 90, and 49 at day 180 (Additional file 1: Table S8).

## Discussion

The LiverT study demonstrated clinically meaningful chronic, and acute, deterioration in liver function following a single TACE in a US cohort of patients with HCC treated in real-world practice. The consistency of this deterioration, using pre-specified thresholds for liver function laboratory parameters, suggests that a sizable proportion of patients in real-world practice do not entirely recover from liver damage after TACE [13].

The robustness of our findings was supported by an additional analysis based on Child–Pugh bilirubin thresholds, which showed a similar proportion of patients with acute and chronic bilirubin deterioration compared with the primary analysis, except for a baseline bilirubin of 2–3 mg/dL. For the group with this relatively modest bilirubin elevation, the proportion of patients with chronic deterioration was highest, suggesting that liver function is relatively fragile in this patient population. Exploratory analysis by baseline ALBI grade also consistently showed deterioration of liver function parameters (except albumin) in both periods, regardless of initial ALBI score. Acute and chronic albumin deterioration was lowest for patients with the worst baseline ALBI scores (grade 3). This difference suggests that an absolute decrease in albumin by ≥ 0.3 g/dL (pre-specified threshold for deterioration) may be less likely to occur when baseline albumin values are already low (i.e. patients with ALBI score > − 1.39; grade 3).

In an interim analysis of the prospective, observational OPTIMIS study in non-US patients, deterioration of bilirubin and albumin following TACE was demonstrated in 14 and 25% of patients, respectively [14]. Although this is lower than in LiverT, the patient population may have differed due to inclusion criteria, regional variation in HCC risk factors, and differences in liver dysfunction reporting [14]. Moreover, experience performing selective TACE (associated with less liver adverse events compared to lobar TACE) may be higher in centers selected for prospective studies compared with those in which our real-world cohort were treated; however, in this study, it was not possible to obtain data on TACE selectivity. Although retrospective studies have also reported deterioration in liver function after TACE [15–17], liver-related abnormalities were not reported for acute versus chronic time points after TACE. Thus, LiverT may provide further insight into the time between TACE and occurrence of liver function deterioration in real-world patients.

Several clinical trials have demonstrated liver function deterioration after TACE; however, our real-world findings may differ due to a more heterogeneous patient population and more variable TACE experience [4, 18–21]. Both factors may have contributed to the higher rate of death in LiverT than generally reported in TACE clinical trials, suggesting a considerable number of real-world TACE treated HCC patients had worse outcomes.

In the phase 2, randomized, double-blind, placebo-controlled SPACE trial, liver function deterioration following TACE plus placebo was low; hyperbilirubinemia was only reported in 9% of patients [19]. Unlike LiverT, SPACE only included patients with measurable HCC lesions, no MVI or distant metastases, and adequate liver function [19]. However, in our study, baseline values were highly variable and, occasionally, would not have met clinical trial inclusion criteria. Additionally, some patients had distant metastases (6%) and PVT (5%) at baseline, both of which are relative contraindications for TACE [22]. As a reflection of real-world clinical practice of TACE, our results highlight the need for appropriate and accurate patient selection to minimize the risk of chronic hepatic dysfunction following TACE [23].

As with all observational, retrospective studies, a number of limitations were unavoidable and should be outlined and discussed. Limitations include potential sampling bias and confounding. Here, we leveraged a national dataset populated with International Classification of Disease codes and structured laboratory data; however, data source-related limitations include potential absences, misclassifications from coding errors, and lack of patient records from which relevant data can be abstracted, such as physician-documented Child–Pugh score, and the size and number of tumors. Missing additional data included laboratory values needed for the analysis of the primary endpoint, which could lead to an underestimation of liver function deterioration. For example, a patient with a mild elevation in AST may have had a severe increase in serum bilirubin; however, if only AST was recorded in the database, liver deterioration could have been underreported. Important TACE procedural information was also unavailable, including the selectivity of the TACE procedure. There is evidence that a more selective approach to delivering TACE (i.e. segmental) leads to less liver damage and better outcomes [24], but data were insufficient to verify whether greater chronic liver damage was associated with non-selective procedures. At the time of analysis, a large, longitudinal HCC database with structured and unstructured data was not readily available. However, 2010 was selected as the starting year for the study because it was assumed that enough time had passed from the demonstration of the superiority of selective TACE [24], and that this procedure would have been adopted as standard practice. Additionally, data were not available to stratify patients according to the degree of tumor burden and stage, similar to many other retrospective studies in this field. Thus, our data source represented the best compromise for evaluation of a relatively large cohort of real-world HCC patients. Additionally, the study only included patients who did not receive additional HCC treatment within 3 months after index TACE. While absence of additional therapy minimized confounding, it may have biased the cohort towards sicker patients by excluding those without post-TACE liver dysfunction who required subsequent treatment in the short term. This bias towards sicker patients may explain the higher percentage of liver function deterioration and mortality compared with other clinical studies. In addition, excluding patients because of a lack of laboratory data may also have contributed to selection bias because it could be implied that patients included in the analysis were more closely followed and monitored for clinical reasons, which could impact liver function. In addition, patients who experienced severe deterioration, leading to death before any further chronic reassessment, would also not have been included. A proportion of patients included in the analyses were treated with TACE despite having distant metastases (6%) and/or PVT (5%), both of which are contraindications for TACE. Lastly, there were no control groups included in the study, such as MELD-matched patients without HCC, who could have demonstrated prevalence of liver function deterioration over a 3-month period without the concomitant effect of liver-directed therapy. Despite these limitations, we believe our cohort is sufficiently representative of real-life situations after TACE to provide insights on the risk of acute and chronic liver dysfunction.

## Conclusions

In summary, these results demonstrate the occurrence of acute and chronic deterioration of liver function following a single TACE treatment in a modern cohort of US HCC patients. The data also suggest that, for a proportion of real-life patients, TACE can be associated with chronic liver function deterioration. The use of a range of different systemic therapies (targeted therapy, anti-programmed cell death 1/ligand-1 treatment, and others) after TACE is increasing following numerous positive survival results in HCC patients with relatively well-preserved liver function [23, 25]. Liver dysfunction may preclude such systemic therapy options. Therefore, the present findings highlight the need for the careful selection of patients for TACE to help optimize the benefit of the overall HCC treatment course.

## Additional file

Additional file 1: This file includes additional results such as figures and tables. (DOCX 60 kb)

## Data Availability

Availability of the data underlying this publication will be determined according to Bayer’s commitment to the European Federation of Pharmaceutical Industries and Associations (EFPIA) and Pharmaceutical Research and Manufacturers of America (PhRMA) “Principles for responsible clinical trial data sharing”. This pertains to scope, time point, and process of data access. As such, Bayer commits to sharing upon request from qualified scientific and medical researchers patient-level clinical trial data, study-level clinical trial data, and protocols from clinical trials in patients for medicines and indications approved in the United States (US) and European Union (EU) as necessary for conducting legitimate research. This applies to data on new medicines and indications that have been approved by the EU and US regulatory agencies on or after January 01, 2014. Interested researchers can use www.clinicalstudydatarequest.com to request access to anonymized patient-level data and supporting documents from clinical studies to conduct further research that can help advance medical science or improve patient care. Information on the Bayer criteria for listing studies and other relevant information is provided in the “Study sponsors section” of the portal. Data access will be granted to anonymized patient-level data, protocols, and clinical study reports after approval by an independent scientific review panel. Bayer is not involved in the decisions made by the independent review panel. Bayer will take all necessary measures to ensure that patient privacy is safeguarded.

## Abbreviations

ALBI: Albumin–bilirubin
ALT: Alanine transaminase
AST: Aspartate transaminase
CI: Confidence interval
CTCAE: Common Terminology Criteria for Adverse Events
HBV: Hepatitis B virus
HCC: Hepatocellular carcinoma
HCV: Hepatitis C virus
INR: International normalized ratio
MVI: Macrovascular invasion
PVT: Portal vein thrombosis
TACE: Transarterial chemoembolization
TARE: Transarterial radioembolization
Y90: Yttrium-90
