# Supplementary material for: Liver function changes after transarterial chemoembolization in US hepatocellular carcinoma patients: the LiverT study
Source: BMC Cancer. 2019 Aug 13;19:795. doi: 10.1186/s12885-019-5989-2 (PMC6693268; doi:10.1186/s12885-019-5989-2)
Supplement: Supplementary file 1 — This file includes additional results such as figures and tables. (DOCX 60 kb) [file 12885_2019_5989_MOESM1_ESM.docx]

**Supplementary materials**

**TABLE S1** Procedural coding used by Optum’s database for TACE analysis

| **Inclusion criteria** | **Code(s)** |
| --- | --- |
| Diagnosis of HCC at least 1 year prior to index TACE and/or TARE | ICD-9 code 155.0 or ICD-10 C22.0 |
| Received at least one TACE:  According to CPT code  According to ICD-9 codes  No presence of Y90 on the same day | 37204, 37243, 75894, 75896, 75898  38.80 or 38.86  HCPCS, C2616, or ICD-9 procedure code 92.28 |
| One of the following laboratory values within 30 days before and between 30 and 90 days after first TACE:  Serum total bilirubin  Serum albumin  ALT  AST  INR | LOINC codes: 1975-2, 14631-6  LOINC codes: 1751-7, 43712-9  LOINC code: 1742-6  LOINC code: 1920-8  LOINC codes: 34714-6, 6301-6 |
| **Exclusion criteria** | **Code(s)** |
| Received any of the following treatments within 3 months of first TACE:  Repeated TACE  Radiofrequency ablation  Percutaneous ethanol injection  Liver resection  Liver transplantation  Chemotherapy^a^  Sorafenib  Radioembolization by Y90 | CPT codes: 37204, 37243, 75894, 75896, 75898  ICD-9 codes: 38.80 or 38.86  ICD-9 code: E926.0  ICD-9 code: 50.24  ICD-9 codes: 50.22, 50.03, 50.23, 50.25, 50.26, 50.0  ICD-9 code: 50.5  NDC codes  NDC code: 50419-488  ICD-9: 92.28 and HCPCS: C2626 |

^a^CPT codes for chemotherapy were ignored if they occurred on the day a patient had TACE because the codes could reflect TACE procedure instead of chemotherapy.

Optum, a division of UnitedHealth Group (Minnetonka, MN), comprises several health data and information companies, providing an integrated database of healthcare claims data combined with a longitudinal electronic health record database housed by Humedica

*ALT* alanine transaminase, *AST* aspartate transaminase, *CPT* Current Procedural Terminology, *HCC* hepatocellular carcinoma, *ICD* International Classification of Diseases, *HCPCS* Healthcare Common Procedure Coding System, *INR* international normalized ratio, *LOINC* Logical Observation Identifiers Names and Codes, *TACE* transarterial chemoembolization, *TARE* transarterial radioembolization, *Y90* yttrium-90

**TABLE S2** The median intervals between day 30 after TACE to the worst value and the last value for the chronic period

| **Value** | ***N*** | **Median time from day 30 post-TACE to worst, days** | **Median time from day 30 post-TACE to last,  days** |
| --- | --- | --- | --- |
| Bilirubin | 462 | 25.0 | 35.5 |
| Albumin | 442 | 20.0 | 36.0 |
| AST | 446 | 24.5 | 35.0 |
| ALT | 441 | 25.0 | 35.0 |
| INR | 251 | 27.0 | 37.0 |

Chronic period, 30–90 days after TACE

*ALT* alanine transaminase, *AST* aspartate transaminase, *INR* international normalized ratio

**TABLE S3** Patient attrition for TACE analyses

|  | **Number of patients^a^** | **%** |
| --- | --- | --- |
| One TACE between January 1, 2010 and March 31, 2016; age ≥ 18 years with HCC diagnosis within 1 year prior to the index TACE | 3963 | 100 |
| No previous TACE within 1 year of index TACE | 3934 | 99.3 |
| Patients without any of the exclusion criteria within 3 months after index TACE | 2821 | 71.2 |
| Patients without a Y90 TARE on index date | 2723 | 68.7 |
| At least one of the six laboratory values within 30 days prior to index TACE | 1314 | 33.2 |
| At least one of the six laboratory values between 30–90 days after index TACE | 806 | 20.3 |
| Patients with laboratory values within 29 days after TACE | 572 | 14.4 |

^a^Electronic health records and claims combined

*TACE* transarterial chemoembolization, *TARE* transarterial radioembolization, *Y90* yttrium-90

**TABLE S4** Acute and chronic liver deterioration following TACE according to baseline ALBI grade

|  | **Baseline ALBI grade** | | | | | |
| --- | --- | --- | --- | --- | --- | --- |
|  | **Grade 1 (≤ -2.60)** | | **Grade 2  (> -2.60 – ≤ -1.39)** | | **Grade 3 (> -1.39)** | |
|  | ***N*** | **Deterioration, n (%)** | ***N*** | **Deterioration,  n (%)** | ***N*** | **Deterioration,  n (%)** |
| **Acute liver deterioration** | | | | | | |
| Bilirubin increase of ≥ 50% | 70 | 20 (29) | 264 | 80 (30) | 106 | 30 (28) |
| Albumin decrease by  ≥ 0.3 g/dL | 70 | 48 (69) | 264 | 140 (53) | 106 | 40 (38) |
| AST increase of > 25% | 68 | 29 (43) | 259 | 112 (43) | 103 | 48 (47) |
| ALT increase of > 25% | 70 | 32 (46) | 262 | 102 (39) | 104 | 52 (50) |
| INR increase of ≥ 25% | 35 | 10 (29) | 123 | 20 (16) | 67 | 22 (33) |
| **Chronic liver deterioration** | | | | | | |
| Bilirubin increase of ≥ 50% | 70 | 14 (20) | 264 | 53 (20) | 106 | 33 (31) |
| Albumin decrease by  ≥ 0.3 g/dL | 70 | 35 (50) | 264 | 84 (32) | 106 | 19 (18) |
| AST increase of > 25% | 68 | 22 (32) | 259 | 84 (32) | 103 | 26 (25) |
| ALT increase of > 25% | 70 | 22 (31) | 262 | 70 (27) | 104 | 18 (17) |
| INR increase of ≥ 25% | 35 | 4 (11) | 123 | 15 (12) | 67 | 14 (21) |

*ALBI* albumin–bilirubin, *ALT* alanine transaminase, *AST* aspartate transaminase, *INR* international normalized ratio, *TACE* transarterial chemoembolization

**TABLE S5** Acute and chronic liver deterioration following TACE according to hepatocellular carcinoma etiology and absence of portal vein thrombosis

|  | **HBV** | | **HCV** | | **Alcoholic cirrhosis** | | **Absence of PVT** | |
| --- | --- | --- | --- | --- | --- | --- | --- | --- |
|  | ***N*** | **Deterioration, n (%)** | ***N*** | **Deterioration, n (%)** | ***N*** | **Deterioration, n (%)** | ***N*** | **Deterioration, n (%)** |
| **Acute liver deterioration** | | | | | | | | |
| Bilirubin increase of  ≥ 50% | 33 | 10 (30) | 181 | 50 (28) | 102 | 31 (30) | 437 | 128 (29) |
| Albumin decrease by  ≥ 0.3 g/dL | 33 | 17 (52) | 180 | 83 (46) | 96 | 45 (47) | 417 | 215 (52) |
| AST increase of > 25% | 34 | 14 (41) | 173 | 65 (38) | 99 | 46 (46) | 421 | 184 (44) |
| ALT increase of > 25% | 33 | 12 (36) | 176 | 61 (35) | 97 | 41 (42) | 416 | 174 (42) |
| **Chronic liver deterioration** | | | | | | | | |
| Bilirubin increase of  ≥ 50% | 33 | 4 (12) | 181 | 50 (28) | 102 | 25 (25) | 437 | 93 (21) |
| Albumin decrease  ≥ 0.3 g/dL | 33 | 12 (36) | 180 | 50 (28) | 96 | 26 (27) | 417 | 129 (31) |
| AST increase of > 25% | 34 | 12 (35) | 173 | 51 (29) | 99 | 32 (32) | 421 | 126 (30) |
| ALT increase of > 25% | 33 | 9 (27) | 176 | 44 (25) | 97 | 23 (24) | 416 | 103 (25) |

*ALT* alanine transaminase, *AST* aspartate transaminase, *HBV* hepatitis B virus, *HCV* hepatitis C virus, *PVT* portal vein thrombosis, *TACE* transarterial chemoembolization

**TABLE S6** Acute and chronic liver deterioration following TACE according to diabetes status

|  | **Diabetic** | | **Non-diabetic** | |
| --- | --- | --- | --- | --- |
|  | ***N*** | **Deterioration, n (%)** | ***N*** | **Deterioration, n (%)** |
| **Acute liver deterioration** | | | | |
| Bilirubin increase of ≥ 50% | 150 | 45 (30) | 312 | 93 (30) |
| Albumin decrease  ≥ 0.3 g/dL | 145 | 76 (52) | 297 | 153 (52) |
| AST increase of > 25% | 147 | 83 (56) | 299 | 111 (37) |
| ALT increase of > 25% | 145 | 74 (51) | 296 | 114 (39) |
| **Chronic liver deterioration** | | | | |
| Bilirubin increase of ≥ 50% | 150 | 32 (21) | 312 | 73 (23) |
| Albumin decrease  ≥ 0.3 g/dL | 145 | 40 (28) | 297 | 99 (33) |
| AST increase of > 25% | 147 | 57 (39) | 299 | 78 (26) |
| ALT increase of > 25% | 145 | 46 (32) | 296 | 64 (22) |

*ALT* alanine transaminase; *AST* aspartate transaminase; *TACE* transarterial chemoembolization

**TABLE S7** INR deterioration in the acute and chronic periods after TACE stratified by anticoagulant use

|  | **Anticoagulant^a^** | | **No anticoagulant** | |
| --- | --- | --- | --- | --- |
|  | ***N*** | **Deterioration, n (%)** | ***N*** | **Deterioration, n (%)** |
| **Acute liver deterioration** | | | | |
| INR increase of ≥ 25% | 101 | 36 (36) | 150 | 26 (17) |
| **Chronic liver deterioration** | | | | |
| INR increase of ≥ 25% | 126 | 26 (21) | 125 | 11 (9) |

^a^Patients were administered or given a prescription for an anticoagulant between the index TACE and INR test. Anticoagulants selected for analysis included rivaroxaban, warfarin, dabigatran, apixaban, heparin, enoxaparin, and tinzaparin

*INR* international normalized ratio, *TACE* transarterial chemoembolization

**TABLE S8** Number of observed death events following TACE

| **Time by which deaths have occurred, days** | **Number at risk** | **Observed death events** | **Cumulative death events** |
| --- | --- | --- | --- |
| 0 | 572 | 0 | 0 |
| 30 | 568 | 4 | 4 |
| 90 | 501 | 35 | 39 |
| 180 | 401 | 49 | 88 |

*TACE* transarterial chemoembolization
